# Biological Aging and Uterine Fibrosis in Cattle: Reproductive Trade-Offs from Enhanced Productivity

**DOI:** 10.3390/cells14130955

**Published:** 2025-06-22

**Authors:** Yuta Matsuno, Kazuhiko Imakawa

**Affiliations:** Research Institute of Agriculture, Tokai University, Kumamoto 862-8652, Japan; imakawa.kazuhiko.s@tokai.ac.jp

**Keywords:** reproductive tract, uterus, senescence, senescence-associated secretory phenotype (SASP), biological aging, fibrosis, ruminants, cattle

## Abstract

Reproductive efficiency in cattle remains sub-optimal, with pregnancy rates often below 50%, despite fertilization rates approaching 100%, indicating that implantation failure and/or early embryonic loss are major limiting factors. This disparity highlights the need to understand the biological and physiological mechanisms underlying implantation failure. This review elucidates the cellular and molecular mechanisms underlying reduced pregnancy rates, with a particular focus on biological aging and fibrosis in the reproductive organs as emerging contributors to uterine dysfunction. Accumulated evidence suggests that metabolic demands associated with intensive breeding strategies aimed at maximizing meat and milk productivity may induce multiple forms of stress, including oxidative stress, metabolic stress, and inflammation, which accelerate biological aging and fibrosis in the female reproductive tract. However, the direct molecular mechanisms remain poorly characterized. We hypothesize that biological aging and fibrosis are interconnected mechanisms contributing to impaired uterine function, resulting in reduced implantation rates. By summarizing recent findings and adopting a comparative perspective, this review explores the extent to which insights from human and mouse models can be applied to cattle, considering species-specific reproductive physiology and metabolic adaptations. It explores their relevance to reproductive inefficiencies and discusses potential strategies to enhance fertility and extend bovine reproductive longevity.

## 1. Introduction

Pregnancy represents a profound physiological transformation in eutherian mammals, requiring adaptive processes across the metabolic, endocrine, and immune systems. Key modifications, such as the structural remodeling of reproductive tissues, hormonal changes, and immunological adaptations, are intricately coordinated and essential for successful implantation, placental development, and maintaining maternal health and fetal growth. However, disruptions in uterine remodeling, hormonal balance, or immune regulation can result in implantation failure, fetal growth restriction, gestational complications, or delays to establishing pregnancy.

Despite extensive research in cattle reproduction, pregnancy rates remain below 50%, despite fertilization rates approaching 100%. Research on cattle reproduction consistently reports fertilization rates approaching 90–100% for heifers, beef, and moderate-yielding dairy cows, though rates may be lower in high-producing dairy cows [1,2,3]. However, pregnancy rates often fall below 50% due to significant embryonic losses. Early embryonic mortality, occurring within the first 16 days post breeding, is the primary factor contributing to pregnancy failure [1,4]. A meta-analysis of beef cattle reproduction found that by day 32 of gestation, 47.9% of cows inseminated were not pregnant [5]. This disparity highlights the need to investigate biological events such as uterine receptivity, embryo–maternal interaction, and metabolic regulation, all of which may contribute to early pregnancy failure and sub-optimal reproductive outcomes.

Accumulated evidence suggests that the cumulative effects of these physiological changes contribute to biological aging of female reproductive organs. Biological aging is associated with decreased fertility and increased pregnancy complications, accompanied by cellular senescence [6], chronic inflammation [7], and altered hormonal balance [8], often leading to fibrosis, particularly in the ovaries and the uterus. Fibrosis, characterized by excessive accumulation of extracellular matrix (ECM) proteins, disrupts normal reproductive function and contributes to infertility in humans and mice [9,10,11,12]. While studies using mouse and human models have provided valuable insights, it remains to be determined whether these findings can be directly applied to cattle, particularly given their ruminant physiology and production context. Furthermore, the applicability of these insights extends beyond fundamental research; whether current therapeutic strategies developed for humans, primates, and mice can be effectively translated to cattle remains inconclusive.

Excessive energy intake disrupts metabolic homeostasis, leading to oxidative stress and chronic inflammation, both of which are key drivers of biological aging [13,14]. In cattle, metabolic stress induced by high-energy feeds and intensive selective breeding for increased milk or meat production has been implicated in accelerating biological aging [15]. Similarly, high-yield selection and intensive breeding practices prioritize productivity over physiological balance, which can impair ECM remodeling in reproductive organs. Impaired ECM remodeling promotes excessive collagen deposition, reducing tissue elasticity and predisposing the uterus to fibrosis. These disruptions may increase metabolic strain and exacerbate tissue fibrosis. Increased dairy milk yield per cow has been associated with chronic nutrient depletion and hormonal imbalances, which may influence epigenetic modifications and have been implicated in the development of uterine fibrosis, potentially affecting reproductive efficiency.

Despite recent fertility-focused breeding indices and protocols, such as the Double Ovsynch, that have improved estrous synchronization and fertilization rates [16,17,18,19], overall reproductive success remains suboptimal, with pregnancy rates in dairy cows still below 50% [20]. This suggests that existing reproductive management protocols may enhance biological aging and compromised uterine function under intensive production practices.

While postpartum uterine infections and hormonal imbalances are well-recognized factors contributing to prolonged calving intervals in dairy and beef cows, an increasing number of cows exhibit extended calving intervals in the absence of apparent infections or endocrine dysfunction. This observation suggests that subclinical processes, such as biological aging and progressive uterine fibrosis, may play a hidden role in compromising uterine recovery and function, particularly under intensive production systems.

In this review, we explore the role of biological aging and uterine fibrosis as underrecognized, yet potentially critical contributors to declining fecundity in cattle. By adopting a comparative perspective from livestock and biomedical research, we aim to provide a comprehensive understanding of the interplay between biological aging and fibrosis in the bovine uterus. Furthermore, we discuss how these biological processes affect the balance between production efficiency and fecundity, highlighting key knowledge gaps and future research directions for improving fertility and sustaining productivity in cattle.

## 2. Biological Aging

### 2.1. Biological Aging and Uterine Function and Infertility

**Figure 1:**
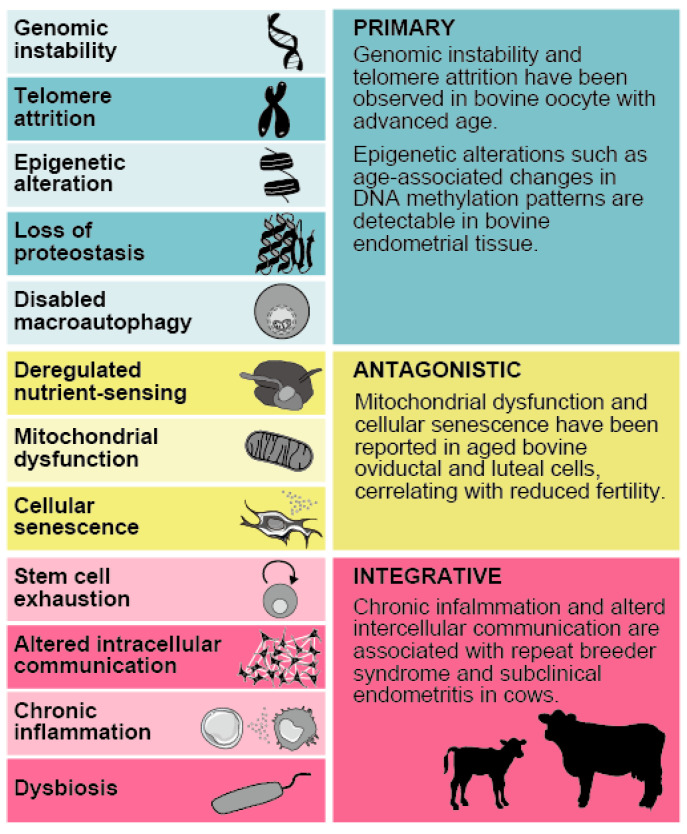
The hallmarks of biological aging categorized into primary, antagonistic, and integrative groups, as proposed by López-Otín et al. (2023) [24]. The 12 key hallmarks of biological aging: genomic instability, telomere shortening, epigenetic modifications, impaired proteostasis, dysfunctional macroautophagy, disrupted nutrient sensing, mitochondrial impairment, cellular senescence, stem cell depletion, altered intercellular signaling, chronic inflammation, and microbiome imbalance. Examples of cattle-specific relevance are noted where available: Genomic instability and telomere attrition have been observed in bovine oocytes with advancing age. Epigenetic alterations such as age-associated changes in DNA methylation patterns are detectable in bovine endometrial tissue. Mitochondrial dysfunction and cellular senescence have been reported in aged bovine oviductal and luteal cells, correlating with reduced fertility. Chronic inflammation and altered intercellular communication are associated with repeat-breeder syndrome and subclinical endometritis in cows. Source: adapted from [24].

Biological aging is characterized by progressive dysfunction in cells, tissues, and organs [21]. In 2023, twelve molecular, cellular, and systemic hallmarks of biological aging were consolidated based on accumulating evidence: genomic instability, telomere attrition, epigenetic alterations, loss of proteostasis, disabled macroautophagy, deregulated nutrient-sensing, mitochondrial dysfunction, cellular senescence, stem cell exhaustion, altered intercellular communication, chronic inflammation, and dysbiosis. These hallmarks fall into three categories: primary, antagonistic, and integrative [22,23,24] (Figure 1). Primary hallmarks are the initial triggers of cellular damage, including genomic instability, telomere attrition, and epigenetic alterations. Antagonistic hallmarks represent responses to these kinds of damage, such as cellular senescence and mitochondrial dysfunction, that are initially protective but become deleterious if persistent. Integrative hallmarks are the consequences of accumulated damage and compensatory failure, such as stem cell exhaustion and altered intercellular communication, which directly impact tissue and organ function. This hierarchical model provides a conceptual framework to understand how biological aging-related processes, once triggered, can converge to impair tissue homeostasis, including uterine function.

These hallmarks play critical roles in reproductive organs, where age-related changes can directly influence fertility. Researchers have increasingly used mouse models to elucidate their roles [25]. Uterine biological aging affects various components, including the myometrium, neurofibers, and endometrium, leading to poorer reproductive performance in mice, rats, and women [23,26]. Recent findings from mouse models suggest that these changes are mediated by altered genetic and epigenetic mechanisms, metabolic conditions, immune system dynamics, and other components of the female reproductive tract [23].

In cattle, several studies have shown that reproductive efficiency decreases with age. The productive lifespan of dairy cattle typically peaks between 3.5 and 5 years, after which fertility begins to decline. Cows aged 7 years or older tend to exhibit significantly reduced reproductive efficiency [27]. A study on Friesian Holstein dairy cattle in Indonesia (2017–2018) reported that, as cows aged from 4 to 5 years, the number of services per conception increased from 3.2 ± 1.8 to 4.3 ± 1.9 (*p* < 0.05), indicating reduced reproductive efficiency [27]. Additionally, Osoro and Wright (1992) found that pregnancy rates declined from 90% in younger Blue-Gray cows to 83% in Hereford × Friesian cows older than 7 years (*p* < 0.05) [28]. This decline is compounded by parity effects, as fertility tends to decrease with each successive calving until dairy cattle reach physiological adaptation to repeated calving rather than chronological age [29]. Biological aging is also associated with the reduced developmental competence of oocyte in crossbred Hereford cows [30]. Older crossbred Hereford cows exhibit elevated circulating follicle-stimulating hormone (FSH) levels, reduced follicular recruitment, smaller ovulatory follicles, increased plasma estradiol levels, and a tendency toward lower luteal-phase progesterone levels [31].

### 2.2. Cellular Senescence in Uterine Biological Aging

**Figure 2:**
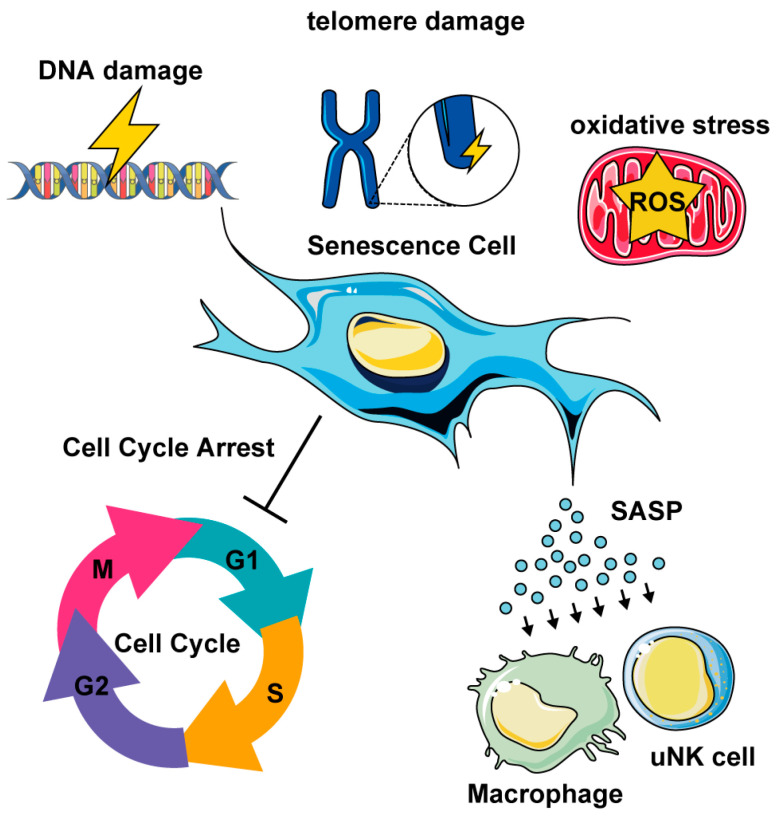
Cellular senescence in the uterus. DNA damage, telomere damage, and oxidative stress drive cellular senescence. Senescent cells show cell cycle arrest and senescent-associated secretory phenotype (SASP). The SASP recruits immune cells, such as macrophages and uterine natural killer (uNK) cells.

Recent studies highlight the role of cellular senescence in uterine biological aging and related infertility in human and mouse models. Cellular senescence is a hallmark of biological aging and is characterized by a state of stable and generally irreversible growth arrest, in which cells permanently cease to divide but remain metabolically active [32,33]. This arrest is triggered by various stresses, such as DNA damage, oxidative stress, and telomere damage [32,33] (Figure 2). Senescent cells exhibit distinct morphological, biochemical, and functional changes, including the senescence-associated secretory phenotype (SASP), which disrupt normal tissue homeostasis and contribute to age-related pathologies [34].

The role of senescent cells in reproductive organs is complex; while senescent cells contribute to ovarian follicle decline and pregnancy complications in human and mouse models [6], they also support embryo/fetal and placental development and parturition through the SASP under normal physiological conditions [35,36]. However, the excessive accumulation of senescent cells is linked to age-related pathologies [37]. For instance, increased senescence and decreased stemness in human endometrial stromal cells during the proliferative phase are associated with implantation failure [38]. Senescent cells in murine ovaries may accelerate reproductive biological aging via SASP [39]. While these findings highlight that cellular senescence has both positive and negative effects on female reproductive biological aging and fertility, the applicability of these observations to cattle remains uncertain and requires careful validation. Cattle exhibit distinct reproductive physiology compared to rodents and primates, such as a 21-day estrous cycle, non-invasive epitheliochorial placentation, and multiple follicular waves per cycle. Therefore, further studies are needed to determine whether senescent cell functions observed in human and mouse models apply similarly to bovine reproductive aging.

Human and mouse models have also revealed that cellular senescence is involved in endometriosis and related infertility. Human endometriotic lesions (consisting of ectopic endometrium), often associated with infertility, exhibit increased expression of senescence markers like p16^Ink4a^ and decreased lamin B1 (*LMNB1*) [40,41]. Oxidative stress in human endometriotic lesions upregulates senescence-associated proteins, contributing to inflammation and infertility [42,43]. The upregulation of Sirtuin 1 (*SIRT1*), a nicotinamide adenine dinucleotide (NAD)-dependent protein deacetylase, promotes epithelial–mesenchymal transition by inducing senescence escape in murine endometriosis [44]. Premature immunosenescence may impair immune surveillance, allowing human endometriotic stem cell migration [45]. During the peri-implantation period, pro-senescent decidual responses have been implicated in recurrent pregnancy loss, further highlighting the interplay between senescence and immune regulation in the human endometrium [46]. Endometrial stromal cellular senescence may contribute to human endometrium dysfunction and implantation failure [47]. Thus, cellular senescence has emerged as a key factor in uterine function and pathology, requiring careful balance to maintain reproductive health.

In cattle, tissue-specific senescence effects have been identified in reproductive organs. Aged oviductal epithelial cells show the upregulation of proinflammatory genes and downregulation of ECM-related genes, accompanied by decreased proliferation rates and lower ciliary activity [48,49,50]. Aged endometrial cells show the activation of inflammation-related and interferon-signaling pathways, accompanied by lower cell viability and proliferation rates [51]. Aged corpus luteum shows decreased progesterone production and secretion, particularly during the early luteal phase [52]. These alterations in bovine reproductive tissues contribute to the overall decline in fertility observed with advanced maternal age in cows.

### 2.3. Molecular Drivers of Biological Aging in the Uterus

Recent studies have demonstrated that several molecular mechanisms are related to biological aging [24]. DNA damage, particularly the accumulation of G-quadruplexes, plays a central role in biological aging by altering gene expression and chromatin structure [53,54]. While reactive oxygen species (ROS) have long been implicated in biological aging, the mammalian target of rapamycin (mTOR) signaling pathway has also emerged as a key regulator of biological aging [55,56]. Non-enzymatic molecular damage, such as advanced glycation end products (AGEs), contributes to biological aging through various spontaneous chemical reactions [57,58]. Telomere shortening and the overall loss of molecular fidelity are also key factors for biological aging and genomic stability [59,60].

In humans, weight gain, obesity, and metabolic stress are associated with accelerated biological aging [61]. Studies have shown that weight gain from early to middle adulthood is linked to higher risks of chronic diseases and decreased odds of healthy biological aging [62]. Obesity and weight gain are inversely associated with telomere length [63]. Metabolic syndrome and associated oxidative stress can contribute to accelerated biological aging [64].

In cattle, inflammaging, which refers to chronic inflammation associated with biological aging, has been implicated in the deterioration of bovine uterine function and age-related reproductive inefficiency [65]. Additionally, while genetic selection has significantly improved both meat and milk yield [66], these achievements have come with unintended consequences, including increased metabolic demands, associated health issues and reduced welfare [67]. This stress includes: altered nutrient metabolism, dysfunctional inflammatory responses, and oxidative stress [68]. Due to the immense metabolic demands of high milk production, high-yielding dairy cows experience a negative energy balance characterized by elevated blood beta-hydroxybutyrate and urea levels [69,70].

How genetic selection interacts with intensive milk production reveals broader biological trade-offs. Intensive breeding practices not only amplify metabolic stress but also accelerate epigenetic aging in dairy cows [15]. This epigenetic aging process impairs bovine immunity, increasing susceptibility to infections such as metritis and endometritis in dairy cows [71]. The demands of milk production also induce endoplasmic reticulum stress in the liver and mammary epithelial cells, compounding health issues and affecting milk yield [72]. Breeding programs focused on milk production have also led to reproduction insufficiencies, such as lower fertility rates and increased calving intervals [73]. The combined effects of genetic selection and intensive milk production highlight the need for sustainable breeding practices.

### 2.4. Epigenetic Aging

Epigenetic aging is defined as age-related changes in DNA methylation, histone modifications, and non-coding RNA (ncRNA) expression, affecting gene regulation and genomic stability, referring to the integrity and maintenance of the DNA structure, without altering the DNA sequence [74,75]. In reproductive organs and tissues, epigenetic aging has profound implications, as it is closely linked to reduced fertility and an increased risk of reproductive cancers in both males and females in human studies [76,77].

In females, oocyte quality declines with age, showing reduced DNA methylation, decreased DNA methyltransferases and histone deacetylase expression, and the loss of heterochromatin marks in human and mouse models [78,79]. These changes contribute to genomic instability, increased DNA damage, and elevated retrotransposon activity in aged oocytes [79].

Epigenetic clocks, which assess biological age based on DNA methylation patterns, provide a robust tool for evaluating reproductive biological aging [80,81,82,83]. Several models, including Horvath’s clock, Hannum’s clock, PhenoAge, and GrimAge [84], have been adapted for ruminants, such as cattle, sheep, and goats to estimate the biological age and assess their reproductive potential [85].

These clocks are valuable for identifying accelerated biological aging linked to health outcomes. Additionally, they can be used to evaluate anti-biological aging interventions and mechanisms [84], although computational refinements are required to enhance their accuracy and utility [86]. Epigenetic aging also relates to nutrient sensing, mitochondrial activity, and stem cell composition, highlighting its complex interactions with metabolism and cellular function [87]. Epigenetic changes related to metabolism, immunity, and autophagy have significant implications for fertility, particularly in bovine reproductive management [88,89].

Emerging tools such as cell-free DNA (cfDNA) in the blood can be used as a minimally invasive biomarker to detect age-associated epigenomic changes [90]. The use of cfDNA could provide insights into inferring age-related epigenetic alterations in the female reproductive tract of cows without the need for direct tissue sampling, offering practical and early interventions, improved breeding strategies, and optimized reproductive management, emphasizing the importance of integrating epigenetic insights into bovine production systems.

### 2.5. Epigenetic Aging in Pregnancy

Recent studies suggest an association between pregnancy and accelerated epigenetic aging in women and mice. For instance, multiple investigations report faster epigenetic aging with an increasing number of giving birth, as measured by epigenetic clocks [91,92,93]. Biomarkers such as telomere shortening and alteration in DNA methylation patterns further support this association [91,93]. Women with preeclampsia exhibit more pronounced epigenetic aging and increased cellular senescence markers compared to normotensive pregnancies [94]. Maternal epigenetic aging during pregnancy is associated with shorter gestation and lower birthweight [95].

In cattle, DNA methylation, among other epigenetic mechanisms, plays a significant role in regulating gene expression in uterine tissues. Analysis of endometrial DNA methylation patterns has identified 1958 differentially methylated CpG sites between pregnant and nonpregnant cows [96]. Additionally, over 1000 significant correlations between endometrial DNA methylation and gene expression have been identified, with 52% showing a negative relationship [97]. Temporal regulation is also observed; for example, 42% of genes exhibiting significant methylation-expression correlations are differentially expressed between pregnant and cycling cows [97]. Furthermore, methionine supplementation alters 3430 CpG sites, potentially influencing metabolic and reproductive pathways via epigenetic regulation [96]. These results suggest that nutritional interventions may modulate epigenetic regulation of endometrial gene networks, potentially influencing reproductive success. These findings have significant implications for bovine breeding, as epigenetic modifications may contribute to biological aging, reduced fertility, and fecundity.

## 3. Fibrosis

### 3.1. Fibrosis in Female Reproductive Organs

**Figure 3:**
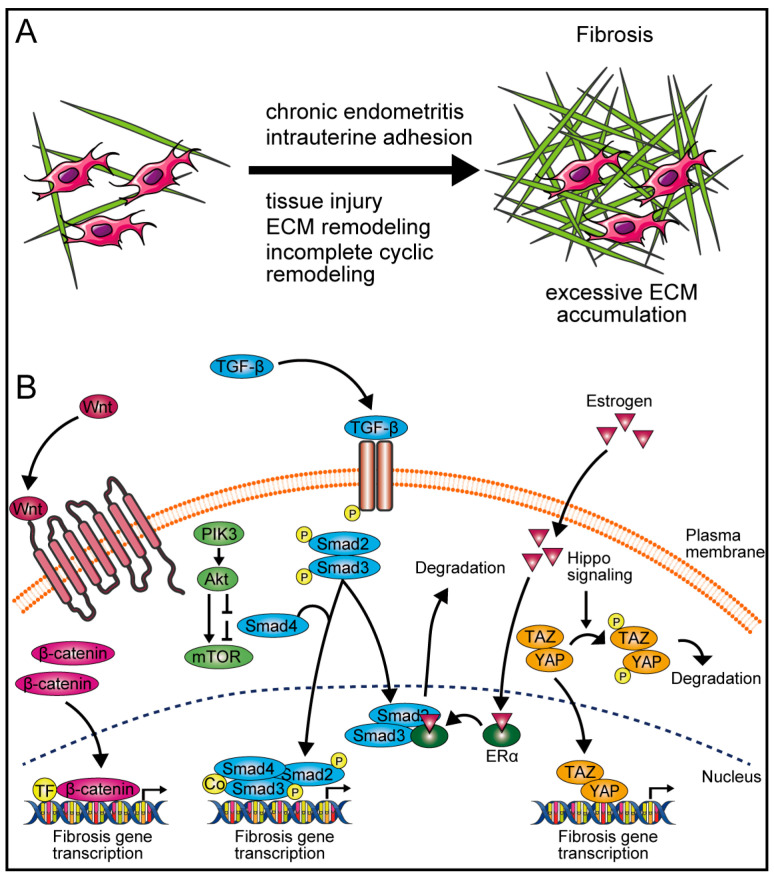
Molecular mechanisms lead to uterine fibrosis. (**A**) Schematic representation of fibrosis progression. This illustration outlines the key cellular and molecular events driving fibrosis development, including excessive ECM accumulation, fibroblast activation, and inflammatory signaling. (**B**) Integrated signaling pathways involved in fibrosis modulation. These include: the wingless-type MMTV integration site family (Wnt)/β-catenin pathway which promotes fibroblast activation and ECM production. Evidence is currently limited to human and rodent models; bovine-specific data are lacking. The transforming growth factor beta (TGF-β) signaling pathway, primarily via the suppressor of mother against decapentaplegic (Smad) cascade, plays a central role in fibrosis by inducing myofibroblast differentiation and collagen synthesis. Validated in bovine and equine endometrium. The phosphatidylinositol-3 kinase (PI3K)-Akt-mammalian target of rapamycin (mTOR) pathway contributes to cell survival, proliferation, and metabolic adaptations that exacerbate fibrosis. There has been no direct validation in cattle uterine tissue, though pathway components are conserved. Estrogen modulates fibrosis by inhibiting the TGF-β/Smad signaling pathway, leading to Smad complex degradation and reduced fibrotic response. This anti-fibrotic role is supported by in vitro studies using bovine endometrial epithelial cells. The Hippo-Yes-associated protein (YAP)/transcriptional coactivator with PDZ-binding motif (TAZ) pathway regulates cellular mechanotransduction and fibrosis-related gene expression, influencing fibroblast activation and tissue remodeling. Established in human uterine fibroid models; not yet validated in bovine uterus. These pathways collectively determine the balance between tissue repair and fibrotic progression. Species-specific differences should be considered when interpreting these molecular interactions in the context of cattle reproduction.

Fibrosis, defined as excessive ECM accumulation, leads to tissue scarring and organ dysfunction, significantly affecting reproductive health (Figure 3A). It commonly occurs in chronic inflammatory diseases and has profound implications for ovarian and uterine physiology. In ovaries, biological aging is characterized by increased fibrosis, which disrupts ovulation, reduces fertility, and increases the risk of ovarian cancer [98,99,100]. This progression involves a combination of chronic inflammation, mitochondrial dysfunction, and altered immune cell populations, contributing to a proinflammatory cellular microenvironment [100,101]. Ovarian fibrosis impairs ovulation events including oocyte release from follicles, follicle rupturing, corpus luteum formation, and post-ovulatory tissue remodeling, critical for maintaining ovarian function in aged mice [102]. Notably, the cytokine signature in human follicular fluid from aged ovaries, characterized by elevated fibroinflammatory markers, correlates inversely with anti-mullerian hormone (AMH) levels, a biomarker of ovarian reserve [103].

Uterine fibrosis develops through multiple interconnected mechanisms. Chronic endometritis has been identified as a key driver, inducing persistent inflammatory responses that promote fibrotic tissue deposition [10]. In humans, ECM remodeling plays a critical role, influencing mechanotransduction pathways that facilitate fibroid growth and uterine structural changes [104,105]. For example, studies of intrauterine adhesions in clinical cases have revealed heightened levels of fibrotic markers and the aberrant activation of stem cell pathways, which lead to infertility and menstrual dysfunction [9,10,11]. In murine models, the repeated ECM remodeling of the reproductive tract, particularly the uterus, oviduct, and vagina by estrous cycles promotes fibrosis accumulation and inflammatory changes due to incomplete resolution of collagen deposition [12]. While uterine fibrosis is linked to surgical interventions in both humans and mice, its etiology also encompasses chronic inflammation, incomplete cyclic remodeling, and tissue injuries [10,12].

In cattle, endometritis can result in uterine fibrosis [106,107,108]. A recent study on first-parity Holstein cows reports that those with early postpartum metritis show increased adenomyosis and uterine fibrosis later in life, suggesting that acute inflammatory events may contribute to persistent pro-fibrosis [109]. Additionally, the lower expression of colony-stimulating factor 2, an embryokine with roles in early pregnancy, has been observed in the endometrial epithelium of older cows, implying the impaired regenerative capacity of the biological aging uterus [110]. Molecular pathways involving transforming growth factor beta (TGF-β)1 signaling via transforming growth factor beta receptor 3 (TGFBR3)/Suppressor of mother against decapentaplegic (Smad) 2/3 have been shown to promote fibrosis in bovine endometrial epithelial cells, although 17β-estradiol may mitigate these effects through G protein-coupled estrogen receptor 1 (GPER1)-mediated mechanisms [108]. Furthermore, collagen-specific chaperone heat shock protein 47 (HSP47), a marker of active collagen synthesis, has been found in higher abundance in the cervix, uterus, and oviducts of aged cows, coinciding with the accumulation of denatured collagen and fibrotic matrix [111,112]. AMH and its receptor (AMHR2) are differentially expressed in reproductive tissues of older cows compared to younger or different breeds, and AMH has been shown to upregulate HSP47 expression in uterine epithelial cells [113,114]. These findings suggest that hormonal signaling pathways may influence the onset or progression of uterine fibrosis. Finally, histopathological studies have highlighted the co-existence of chronic inflammation and fibrosis within the endometrial tissues of repeat breeder cows, further supporting the inflammatory–fibrotic axis in bovine reproductive biological aging [115].

Endometrial biopsies in cattle with reproductive disorders commonly reveal chronic endometritis as the predominant histopathological finding [116,117,118]. Key features include mononuclear leukocyte infiltration, dilated uterine glands, and periglandular fibrosis [117,118]. Subclinical endometritis is frequently observed in repeat breeder cows, with inflammatory cell infiltration, epithelial degeneration, and desquamation [118]. Histopathological lesions can be present in cows with or without clinical signs of reproductive disorders [116]. Epithelial height, segmented cell counts, and inflammatory changes show correlations between uterine horns and across time points [119]. Gland number, dilation, and fibrosis are interrelated, with a negative correlation between glandular characteristics and inflammatory changes [119]. Endometrial biopsy is considered a valuable diagnostic tool for assessing fertility potential in dairy cows [116,118].

Equine endometrial fibrosis is a well-characterized condition that serves as a valuable comparative model for studying uterine fibrosis in cattle. It is defined by progressive endometrial and periglandular fibrosis, a reduction in the uterine glands, and is strongly associated with biological aging [120]. Histologically, it features myofibroblast differentiation and aberrant deposition of ECM proteins, including collagen accumulation outside the basement membrane [121]. Additionally, fibrotic remodeling is not limited to the endometrium, studies have also identified fibrosis and myometrial atrophy in the uterine smooth muscle layer of mares [120], suggesting broader uterine involvement. Proposed mechanisms include vascular compromise, lymphatic edema, and chronic inflammation. Neutrophil extracellular traps, while primarily involved in pathogen defense, have been implicated in the upregulation of fibrosis-associated genes [122]. Pro-fibrotic cytokines, interleukins, and prostaglandins also contribute to fibrogenesis [122]. Understanding the signaling pathways involved in myofibroblast proliferation and ECM remodeling in mares provides mechanistic insights that are translatable to bovine uterine fibrosis.

### 3.2. Mechanisms of Fibrosis

Key signaling pathways, including TGF-β, wingless-type MMTV integration site family (Wnt)/β-catenin, and Hippo, regulate fibrosis development through ECM deposition and myofibroblast differentiation (Figure 3B) [123,124,125]. TGF-β signaling is initiated when ligands bind to type II and type I serine/threonine kinase receptors on the cell surface. This activates the phosphorylation of the receptor-regulated Smad proteins, which form complexes with Smad4 and translocate to the nucleus to regulate gene expression [126]. When the Hippo pathway is activated, mammalian Ste20-like kinase 1/2 (MST1/2) kinases phosphorylate and activate large tumor suppressor 1/2 (LATS1/2), which in turn phosphorylate the Yes-associated protein (YAP) and transcriptional coactivator with PDZ-binding motif (TAZ), leading to their cytoplasmic sequestration or degradation, which prevent its transcriptional activation. When the Hippo pathway is inactive, unphosphorylated YAP/TAZ translocate into the nucleus, where they bind to TEA domain family member (TEAD) transcription factors, promoting the expression of downstream genes [127]. The activation of the Wnt/β-catenin pathway leads to the stabilization and nuclear translocation of β-catenin, promoting the transcription of fibrosis-related genes [128].

These pathways interact extensively, forming a cross-regulation network that amplifies fibrotic responses. For instance, TGF-β signaling enhances β-catenin stabilization, thereby upregulating the transcriptional regulation of ECM components in human fibroid and myometrial cells from hysterectomy surgeries [129]. The Hippo pathway, through YAP/TAZ, further contributes to human fibrosis development [124]. Such interactions illustrate the intricate and dynamic interplay between these pathways in uterine fibrosis development. Additionally, the dual specificity protein phosphatase 4 (DUSP4)/glycogen synthase kinase-3 beta (GSK3β)/snail family transcriptional repressor 1 (SNAI1) pathway, implicated in the human endometrial fibrosis of patients with intrauterine adhesions [130], appears to function downstream of Wnt/β-catenin signaling, suggesting its role as a secondary amplifier of fibrotic gene expression.

Hormonal factors, particularly estrogens, are involved in this cross-regulation. Estrogens modulate ECM dynamics by promoting hypercoagulability and altering fibrin network formation, particularly during the menstrual cycle in women [131,132]. Estrogen signaling plays a crucial role in human fibroid pathobiology, involving both genomic and non-genomic pathways such as mitogen-activated protein kinase (MAPK) and phosphatidylinositol-3 kinase (PI3K)-Akt-mTOR [133]. Moreover, a human breast cancer cell study shows that estrogens can bind to estrogen receptor alpha (ERα), interacting with the Smad complex to facilitate its degradation resulting in the inhibition of TGF-β signaling and thereby exerting anti-fibrotic effects [134]. While most mechanistic insights into estrogen’s anti-fibrotic roles derive from human studies, evidence in cattle has also emerged. Specifically, 17β-Estradiol attenuates TGF-β1-induced fibrosis in bovine endometrial epithelial cells via the GPER1-mediated inhibition of TGFBR3/Smad2/3 signaling [108]. These findings indicate that estrogens may play conserved but potentially species-specific roles in modulating uterine fibrosis. These multifaced actions of estradiol highlight its complex contribution to uterine homeostasis. However, the extent to which this inhibition counterbalances other pro-fibrotic signals, such as those mediated by YAP/TAZ or neurogenic locus notch homolog protein 1 (Notch1), remains unclear. Although Notch1 signaling upregulates collagen expression, promoting fibrosis in mouse uterus [135], the applicability of this finding to cattle remains uncertain. Cross-species differences in reproductive physiology and gene regulation necessitate further studies to validate the role of Notch1 signaling in bovine uterine fibrosis. Activin A, a cytokine belonging to the TGF-β superfamily, plays a role in inflammation, tissue repair, and fibrosis, suggesting its involvement in the complexity of signaling crosstalk associated with uterine fibroid development in reproductive-age women [136]. Understanding the molecular mechanisms underlying uterine fibrosis across species will help researchers develop targeted therapeutic interventions to restore uterine function and enhance fertility.

Recent research highlights fibroblasts as active regulators of inflammation and fibrosis in various organs. In the context of endometrial fibrosis, fibroblasts contribute to pathological tissue remodeling by interacting with immune cells, such as macrophages and T cells, and releasing pro-fibrotic mediators, including TGF-β, interleukin 6 (IL6), and vascular endothelial growth factor (VEGF). This role is particularly pronounced in conditions like endometriosis and intrauterine adhesions [137,138,139]. Myofibroblasts, derived from precursors like endothelial cells via endothelial-to-mesenchymal transition, are key drivers of fibrosis [137]. Single-cell RNA sequencing reveals increased proportions of myofibroblasts, pericytes, and macrophages in human ectopic endometrium, suggesting a coordinated response that contributes to tissue remodeling and fibrosis [140]. Myometrial cells can transition to myofibroblast-like phenotypes, forming collagen- and fibronectin-rich nodules, which are hallmarks of fibrotic progression in uterine tissues [141]. Human endometrial mesenchymal stem cells can differentiate into stromal fibroblasts, with those from endometriosis patients showing progesterone resistance and an inflammatory phenotype [142]. These findings highlight the interplay between immune dysregulation and stromal cell dysfunction in fibrosis progression. Recent studies indicate that immunoglobulin G (IgG) accumulates across aged tissues, such as in the brain, liver, and spleen, in both humans and mice [143], while IgG accumulation has been shown to activate macrophages and induce fibrosis and inflammation in adipose tissue [144]. Understanding these mechanisms is essential for developing targeted therapies for endometrial fibrosis.

The coagulation system, particularly fibrin formation, plays an important role in fibrosis development [145]. However, the relationship between fibrin and fibrosis is complex, involving both protective and pathogenic roles. For instance, fibrin accumulation serves as a scaffold for fibroblast recruitment and ECM deposition, while excessive fibrin degradation can exacerbate inflammation, indirectly promoting fibrosis. While some studies using murine models suggest that fibrin removal is not sufficient to prevent fibrosis [146,147], others indicate that thrombin inhibition can reduce fibrosis in pulmonary and hepatic tissues [148]. The plasminogen activation system also contributes to fibrosis, with plasminogen activator inhibitor-1 (PAI-1) promoting fibrosis by inhibiting fibrinolysis and enhancing ECM stability [149]. Notably, coagulation and fibrinolysis factors are dysregulated in fibrotic diseases such as lung and renal fibrosis in humans, where protease-activated receptor (PAR) signaling and growth factor activation (e.g., TGF-β) play a central role [150,151].

Studies in rat models show that excessive hormone treatment, such as dehydroepiandrosterone (DHEA), can induce uterine fibrosis by promoting coagulation activity and ECM deposition, leading to fibrotic remodeling [152]. However, species-specific differences between rodents and cattle necessitate careful interpretation of these findings in the context of bovine reproductive health.

Notably, coagulation- and fibrinolysis-related factors are highly expressed at the trophoblast–endometrium interface during the implantation period in cattle and sheep, which is essential for ECM remodeling and successful implantation [153,154,155,156]. Disruptions in the balance between coagulation and fibrinolysis may contribute to persistent inflammation and excessive ECM deposition, resulting in uterine fibrosis. Maintaining this balance is important not only for successful implantation but also in preventing pathological fibrosis that could affect reproductive efficiency.

## 4. Future Direction

### 4.1. Targeting Senescent Cells in the Bovine Uterus: Promise and Limitations

Pharmacological approaches, including stem cell therapy, anti-biological aging drugs, and herbal medicines, have been investigated for their ability to influence uterine biological aging. For instance, mesenchymal stem cells have demonstrated the ability to enhance endometrial regeneration through paracrine signaling, while compounds like metformin and resveratrol exhibit anti-senescence effects by decreasing the expression of senescence-related markers, such as p21 and RB transcriptional corepressor 1 (RB1) hypophosphorylation in rodent and human models [22,23].

Senolytic drugs selectively induce apoptosis in senescent cells, which influences female reproductive biological aging and pregnancy outcomes. For example, the Dasatinib and Quercetin combination (D+Q) has shown efficacy in reducing senescent cell burden in murine preclinical models, thereby enhancing tissue function. In mice, F4/80+ macrophages play a role in clearing senescent cells from the postpartum uterus, preserving uterine function [157]. Recent studies in rodents and primates have explored senotherapeutics as a strategy to counteract reproductive biological aging [158]. The development of senolytic treatments could lead to breakthroughs in managing age-related infertility and improving uterine receptivity during the implantation period. However, the translation of senolytic therapies to cattle presents major challenges. Practical considerations include differences in pharmacokinetics due to ruminant digestion, the route and frequency of drug administration in large animals, tissue distribution barriers, and safety concerns. In addition, regulatory hurdles for veterinary use of senolytics in food-producing animals remain substantial. Thus, while these compounds are promising in preclinical models, their application to bovine reproductive medicine requires further validation and adaptation.

Research on eliminating senescent cells in rodents and primates has shown promise. Several strategies target and eliminate these cells. For example, natural killer group 2 member D chimeric antigen receptor (NKG2D-CAR) T cells effectively remove senescent cells in aged mice and nonhuman primates, highlighting the potential of immunotherapy in managing senescence [159]. Similarly, mitochondria-targeted tamoxifen (MitoTam) exploits low adenine nucleotide translocase-2 expression to selectively eliminate senescent cells in mice [160]. These strategies highlight the diversity of potential therapeutic targets, ranging from mitochondrial dysfunction to anti-apoptotic signaling pathways. The inhibition of the anti-apoptotic proteins B-cell lymphoma-w (BCL-W) and B-cell lymphoma-extra-large (BCL-XL) has been shown to induce apoptosis in senescent cells in the lungs of ionizing radiation-exposed mice [161]. However, the potential off-target effects and long-term safety of these interventions necessitate careful evaluation before implementation.

Sodium–Glucose Transport Protein 2 (SGLT2) inhibitors, initially developed for diabetic treatment, exhibit senolytic properties. Canagliflozin, an SGLT2 inhibitor, reduces senescent cells in visceral adipose tissue, extends lifespan in obese and aging mice [162], and enhances immune clearance by downregulating programmed cell death ligand 1 (PD-L1) expression [163,164]. This senolytic activity may stem from its ability to modulate oxidative stress and inflammation through nuclear factor, erythroid derived 2, like 2 (NFE2L2) activation [165]. However, the applicability of SGLT2 inhibitors to cattle remains uncertain due to fundamental differences in glucose metabolism and renal physiology between humans and ruminants. In cattle, glucose homeostasis is primarily regulated through hepatic gluconeogenesis [166], and renal glucose reabsorption mechanisms may differ functionally, potentially affecting both drug efficacy and pharmacokinetics. Further research is necessary to evaluate whether SGLT2 inhibitors exert similar senolytic or anti-inflammatory effects in bovine tissues, and whether their administration would be feasible and safe in a veterinary reproductive context.

Senolytic drugs show promise in treating age-related conditions in human and mouse models [167], but their effects on female reproductive biological aging remain unclear. Treatment with D+Q reduced uterine fibrosis and increased p53 expression in aged mice [168], indicating potential benefits for uterine function. However, these treatments failed to improve ovarian reserve or fertility [39]. In human endometrial stromal cells, D+Q enhanced decidualization and reduced senescence markers, suggesting their role in improving uterine cellular function [169].

Sodium tanshinone IIA sulfonate, a natural compound from a traditional Chinese herbal medicine, reduces lesion development by inducing cellular senescence in a mouse model of deep endometriosis [170]. This finding highlights the context-dependent effects of senescence, where its induction in pathological contexts may help limit disease progression, in contrast to senescent cell clearance strategies used in other conditions. Although senolytic therapies aim to mitigate uterine dysfunction, their impact on extending reproductive longevity remains uncertain [158].

Recent studies have shown that senescent cells form a heterogeneous population [171,172], highlighting the need for precise elimination strategies targeting senescent cells that contribute to chronic inflammation and hormonal dysregulation, ultimately impairing uterine function and fertility. Emerging approaches such as single-cell transcriptomics and proteomics may aid in identifying unique markers of senescent cell subtypes in reproductive tissues, facilitating the development of precise senolytic therapies. While single-cell transcriptomic approaches are not yet routine in livestock research, they hold promise for identifying bovine-specific senescence markers and tailoring selective interventions. However, current senolytic therapies remain largely experimental in cattle, and future studies must address pharmacokinetics, tissue targeting, and safety under field conditions.

### 4.2. Preventing Fibrosis Development in Female Reproductive Tissues: From Rodents to Ruminants

Fibrosis is a wound-healing response that can become pathogenic if it continues unchecked. Key mechanisms for fibrosis resolution include degradation of ECM, elimination of fibrogenic myofibroblasts through apoptosis, senescence, or reprogramming, and the modulation of inflammatory responses [173,174]. In the liver, hepatic stellate cell (HSC) apoptosis and reduced expression of metalloproteinase inhibitors play important roles in spontaneous fibrosis resolution [175]. Additionally, macrophages contribute through phenotypic switching, which modulates inflammatory responses and promotes tissue remodeling [176]. Targeting HSC activation, inducing HSC apoptosis, or modulating fibrosis-related signaling pathways are promising strategies for promoting liver fibrosis regression [177].

Recent studies highlight the potential of antifibrotic therapies to reverse fibrosis in female reproductive organs. However, direct evidence linking these therapies to improved fertility and reproductive longevity remains limited in mouse models [12,99]. In vitro studies using bovine endometrial cell cultures have provided valuable insights into fibrosis regulation in cattle. Ginsenoside Rg1 alleviates lipopolysaccharide-induced fibrosis of bovine endometrial epithelial cells by inhibiting reactive oxygen species-activated NLR family pyrin domain containing 3 (NLRP3) [107]. Similarly, melatonin mitigates endometrial fibrosis in bovine endometritis by regulating TGF-β/Smad and MAPK signaling pathways via MT2 receptor [106].

Epigenetic aging, associated with altered coagulation and fibrotic remodeling, is thought to play a role in exacerbating fibrosis, yet the precise molecular mechanisms remain under investigation [178,179,180]. In vitro studies have demonstrated that histone deacetylase inhibitors, such as valproic acid and trichostatin A, can suppress fibrotic gene expression and epithelial-to-mesenchymal transition in uterine and hepatic fibroblasts [178,180]. In vivo animal models further support the anti-fibrotic effects of DNA methyltransferase inhibitors and SIRT1 activators in reversing fibrosis-associated gene expression and improving reproductive outcomes [179]. Despite these promising findings, the in vivo efficacy of these interventions in cattle remains unproven. Challenges include: A lack of long-term studies on reproductive outcomes (e.g., pregnancy rate, uterine involution). Limited pharmacokinetic and safety data for repeated use in livestock. Regulatory barriers to implementing molecular therapies in food-producing animals. Thus, while epigenetic and molecular antifibrotic strategies offer mechanistic promise, their applicability in bovine reproductive practice requires careful evaluation through well-designed translational studies, especially those incorporating fertility endpoints relevant to dairy and beef production systems.

### 4.3. Microbiome-Based Strategies to Modulate Bovine Uterine Immunity

Unlike primates and rodents, cattle present unique challenges in applying senolytic drugs directly to modulate uterine biological aging. The rumen microbiome plays a role in nutrient metabolism, immune modulation, and systemic inflammation, offering a potential target for interventions aimed at improving reproductive function. Specifically, short-chain fatty acids such as butyrate, produced by fiber-fermenting bacteria, have been shown to exert anti-inflammatory effects by inhibiting NF-κB signaling and enhancing regulatory T-cell responses, which may indirectly influence uterine immune tone [181,182]. Moreover, certain microbial metabolites can modulate endocrine signaling, such as estrogen metabolism, via enterohepatic circulation, thereby affecting reproductive tissues including the uterus.

Instead of relying solely on direct senolytic treatments, modifying the rumen microbiome or developing targeted delivery strategies may provide a more viable approach to optimizing uterine health in cattle.

Probiotic interventions, particularly intravaginal administration of lactic acid bacteria (LAB), have demonstrated the ability to influence the bovine uterine immune environment through multiple mechanisms [183]. LAB modulate the mucosal immune system by stimulating pattern recognition receptors, including Toll-like receptors, on epithelial and immune cells, leading to altered cytokine production. Specifically, LAB administration reduces local proinflammatory cytokines such as IL-1β, IL-6, and IL-8, while increasing anti-inflammatory cytokines like IL-10 and enhancing secretory immunoglobulin A production [184,185,186]. These immune shifts correlate with lower incidences of uterine infections, such as metritis and Escherichia coli challenge, as well as improvements in reproductive parameters, including earlier estrus expression and increased conception rates [186].

In addition, a microbiome-targeted phytochemical, aloe-emodin, has been shown to enhance resistance to *Staphylococcus aureus* invasion by enhancing tight junction protein expression and reducing endometrial epithelial cell apoptosis [187]. This suggests that modulation of host–microbe interaction at the epithelial level is another plausible mechanism by which microbiome-based interventions exert protective effects on the uterus. These findings collectively suggest that well-designed microbial interventions can beneficially modulate local inflammatory responses in the bovine uterus, potentially improving reproductive outcomes. Compared to systemic drug delivery, microbial and phytochemical interventions offer more practical routes in cattle, such as intravaginal or intrauterine administration. These strategies show advantages for field-level adoption, especially in dairy systems facing postpartum uterine disorders.

## 5. Conclusions

Extensive research in humans, primates, and rodents has provided insights into uterine fibrosis and biological aging. However, the pathophysiological mechanisms underlying uterine biological aging and fibrosis in cows, particularly in high-producing dairy cows, remain insufficiently characterized, and species-specific mitigation strategies have yet to be established. Addressing uterine biological aging and fibrosis in cattle requires an approach that considers species-specific reproductive physiology and the physiological consequences of intensive production systems. Research strategies include (1) nutritional modulation tailored for ruminant digestion and metabolism to alleviate metabolic stress, (2) epigenetic interventions such as SIRT1 activation or histone deacetylase inhibition adapted to bovine uterine tissue, and (3) microbiome-targeted approaches to regulate the immune environment and reduce inflammatory signaling in the bovine uterus. These strategies should be validated in both in vitro and in vivo bovine models to assess their effects on implantation, pregnancy maintenance, and reproductive longevity.

Understanding the mechanisms of biological aging is essential for enhancing reproductive efficiency in cattle. Identifying reliable biological aging markers, such as epigenetic clock, fibrosis levels, and immune alterations, will support the development of targeted interventions. For example, therapeutic strategies that aim to reduce fibrosis or rejuvenate uterine tissue could improve implantation and pregnancy outcomes. Given the strong link between metabolic stress and biological aging, nutritional status must be carefully considered to avoid chronic energy surplus and support endocrine homeostasis. Excessive energy intake, required for high-producing cows, and metabolic dysregulation can accelerate uterine biological aging, promoting fibrosis and impaired fertility.

While the rumen microbiome helps the cattle digest feed to obtain nutrients and maintain overall bovine health, its influence on the uterine environment and reproductive function remain understudied. Investigating the microbial population associated with reproductive health and identifying strategies to manipulate them, either through nutrition or probiotic interventions, may offer a novel approach to support uterine function and fecundity. Recent studies in sheep have shown that increased cervical microbial abundance and diversity are associated with reduced fertility following artificial insemination, suggesting a link between microbial homeostasis and reproductive efficiency in livestock [188]. While these findings provide valuable insights, species-specific validation is needed to determine whether similar microbial mechanisms influence bovine uterine function. A deeper understanding of these interconnected mechanisms could help optimize breeding strategies that mitigate the negative effects of intensive production systems, although significant challenges remain. These include a lack of bovine-specific longitudinal data, variability in microbial responses to interventions, and practical implementation hurdles in commercial farm settings. Ultimately, improving uterine health through integrative approaches, such as metabolic regulation, anti-fibrotic therapies, and microbiome modulation, holds promise, but will require further research and careful field-level validation to support fertility while maintaining production sustainability.

**Figure 4:**
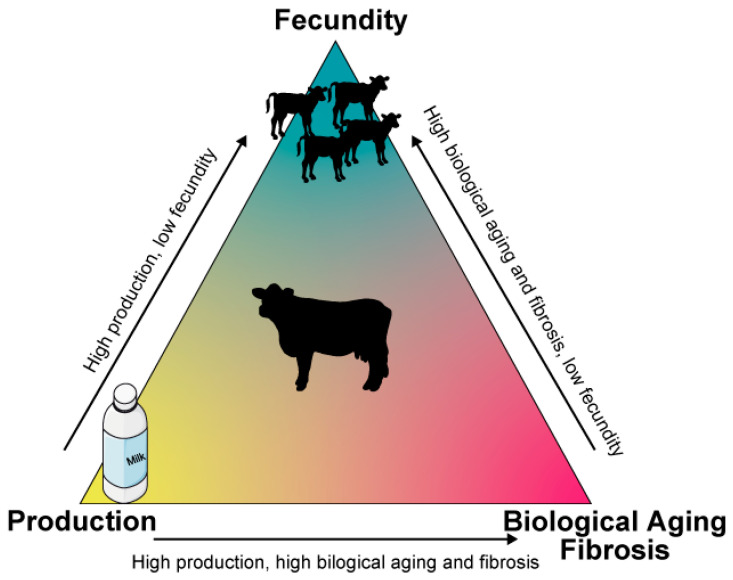
Production–Fecundity Trade-off in Cattle. Higher productivity reduces fecundity due to increased metabolic demands. Higher productivity increases the risk of biological aging and fibrosis. Biological aging and fibrosis contribute to decreased fecundity. It is essential to balance productivity with reproductive health while minimizing biological aging and fibrosis risks.

Advancing knowledge of biological aging and fibrosis is essential to address the reproductive limitations imposed by high productivity demands in modern livestock production, such as fertility management, metabolic stress, and sustainability. Such insights can inform the development of sustainable breeding strategies that balance reproductive longevity with production efficiency. Ultimately, improving uterine health through integrative approaches, including metabolic regulation, anti-fibrotic therapies, and microbial modulation, will support fertility while maintaining production sustainability in modern livestock systems (Figure 4).

## Data Availability

No new data was generated.

## Abbreviations

The following abbreviations are used in this manuscript:

ECM	Extra-cellular matrix
FSH	Follicle-stimulating hormone
SASP	Senescence-associated secretory phenotype
LMNB1	Lamin B1
SIRT1	Sirtuin 1
NAD	Nicotinamide adenine dinucleotide
ROS	Reactive oxygen species
mTOR	Mammalian target of rapamycin
AGEs	Advanced glycation end products
ncRNA	Non-coding RNA
cfDNA	Cell-free DNA
AMH	Anti-mullerian hormone
TGF-β	Transforming growth factor beta
TGFBR3	Transforming growth factor beta receptor 3
Smad	Suppressor of mother against decapentaplegic
GPER1	G protein-coupled estrogen receptor 1
HSP47	Heat shock protein 47
WNT	Wingless-type MMTV integration site family
MST1/2	Mammalian Ste20-like kinase 1 and 2
LATS1/2	Large tumor suppressor 1 and 2
YAP	Yes-associated protein
TAZ	Transcriptional coactivator with PDZ-binding motif
TEAD	TEA domain family member
DUSP4	Dual specificity protein phosphatase 4
GSK3β	Glycogen synthase kinase-3 beta
SNAI1	Snail family transcriptional repressor 1
MAPK	Mitogen-activated protein kinase
PIK3	Phosphatidylinositol-3 kinase
ERα	Estrogen receptor alpha
Notch1	Neurogenic locus notch homolog protein 1
IL6	Interleukin 6
VEGF	Vascular endothelial growth factor
IgG	Immunoglobulin G
PAI-1	Plasminogen activator inhibitor-1
PAR	Protease-activated receptor
DHEA	Dehydroepiandrosterone
RB1	RB transcriptional corepressor 1
D+Q	Dasatinib and Quercetin combination
NKG2-CAR	Natural killer group 2 member D chimeric antigen receptor
MitoTam	Mitochondria-targeted tamoxifen
BCL-W	B-cell lymphoma-w
BCL-XL	B-cell lymphoma-extra-large
SGLT2	Sodium–glucose transport protein 2
PD-L1	Programmed cell death ligand 1
NFE2L2	Nuclear factor, erythroid derived 2, like 2
HSC	Hepatic stellate cell
NLRP3	NLR family pyrin domain containing 3
NF-κB	Nuclear factor kappa-light-chain-enhancer of activated B cells
LAB	Lactic acid bacteria
uNK	uterine natural killer
